# The Autonomous Mind: The Right to Freedom of Thought in the Twenty-First Century

**DOI:** 10.3389/frai.2019.00019

**Published:** 2019-09-26

**Authors:** Simon McCarthy-Jones

**Affiliations:** Department of Psychiatry, Trinity College Dublin, Dublin, Ireland

**Keywords:** human rights, privacy, psychology, law, machine learning, big data

## Abstract

To lose freedom of thought (FoT) is to lose our dignity, our democracy and our very selves. Accordingly, the right to FoT receives absolute protection under international human rights law. However, this foundational right has been neither significantly developed nor often utilized. The contours of this right urgently need to be defined due to twenty-first century threats to FoT posed by new technologies. As such, this paper draws on law and psychology to consider what the right to FoT should be in the twenty-first century. After discussing contemporary threats to FoT, and recent developments in our understanding of thought that can inform the development of the right, this paper considers three elements of the right; the rights not to reveal one's thoughts, not to be penalized for one's thoughts, and not to have one's thoughts manipulated. The paper then considers, for each element, why it should exist, how the law currently treats it, and challenges that will shape it going forward. The paper concludes that the law should develop the right to FoT with the clear understanding that what this aims to secure is mental autonomy. This process should hence begin by establishing the core mental processes that enable mental autonomy, such as attentional and cognitive agency. The paper argues that the domain of the right to FoT should be extended to include external actions that are arguably constitutive of thought, including internet searches and diaries, hence shielding them with absolute protection. It is stressed that law must protect us from threats to FoT from both states and corporations, with governments needing to act under the positive aspect of the right to ensure societies are structured to facilitate mental autonomy. It is suggested that in order to support mental autonomy, information should be provided in autonomy-supportive contexts and friction introduced into decision making processes to facilitate second-order thought. The need for public debate about how society wishes to balance risk and mental autonomy is highlighted, and the question is raised as to whether the importance attached to thought has changed in our culture. The urgency of defending FoT is re-iterated.

“*Carefully guard your thoughts because they are the source of true life*.”

Proverbs 4:23

“*[I]t is proper to take alarm at the first experiment on our liberties… The freemen of America did not wait till usurped power had strengthened itself by exercise, and entangled the question in precedents. They saw all the consequences in the principle, and they avoided the consequences by denying the principle*.”

James Madison (1785)

To lose sovereignty over our minds is to lose our dignity, our democracy, and even our very selves. Such sovereignty is termed mental autonomy. This is “the specific ability to control one's own mental functions,” which include attention, memory, planning, rational thought and decision making (Metzinger, 2013). Dignity, “the presumption that one is a person whose actions, thoughts and concerns are worthy of intrinsic respect, because they have been *chosen, organized and guided*” (Nuffield Council on Bioethics, 2002, p. 121, italics added) requires mental autonomy. Democracies, in which citizens choose the laws that bind them (Johnson and Cureton, 2019), are only possible if citizens are mentally autonomous. The ability to think freely is so essential to our identity that to violate it is to deprive us “of personhood altogether” (Halliburton, 2009, p. 868).

Law must hence safeguard mental autonomy. International human rights law does this through the right to freedom of thought (FoT) (Nowak, 1993). In the United States, FoT is protected by the Bill of Rights (Blitz, 2010; Richards, 2015). Given the centrality of the right to FoT to both personhood and democracy, one may expect it to be clearly defined and frequently exercised. One would be wrong in both cases.

The right to FoT is poorly defined. Attempts to sketch its contours have been negligible (Kolber, 2016). It is unclear what counts as thought, what qualifies as a violation of the right, whether the right should be absolute and, if not, what would justify its violation (Mendlow, 2018). As a result, the right is rarely invoked. In the United States its exercise most often involves cases of sexual thoughts about minors (e.g., *U.S. v. Gamache*, 1998; *Doe v. City of Lafayette, Indiana*, 2003; *U.S. v. Bredimus*, 2003; *U.S. v. Kaechele*, 2006; *U.S. v. Tykarsky*, 2006; *U.S. v. Stokes*, 2013; for a related case see *State of Washington v. Stevenson*, 2005). Has the right to FoT, a foundational human right, really degenerated into merely the right to think the unspeakable?

Nineteenth-century technological advances spurred legal resistance that yielded the new conception of a right to mental privacy (Warren and Brandeis, 1890). Today, twenty-first century technological advances pose new threats to FoT. These demand we clearly draw the contours of the right to FoT to ensure our mental autonomy in this new landscape. This process needs to be informed by developments in the psychological understanding of thought. As such, this paper will consider, in an interdisciplinary manner, what the right to FoT should be in the twenty-first century.

## Challenge I: New Technological Abilities to Access Thought

### Behavior-Reading

Humans bleed data. For the longest time, it seeped into the earth where it fell or was washed away by the tides of time. Now, new technologies capture and store it indefinitely. Technology behemoths, such as Facebook and Google, possess unprecedented troves of user data. Data-analytic companies hold thousands of data points on millions of people (e.g., i360, 2019). National security agencies can access even greater volumes of data (Greenwald, 2013).

This data can now be analyzed, using advanced machine learning algorithms, to infer unobservable inner states of individuals. This can be termed “behavior-reading.” People's observable behaviors, including their facial expressions, actions, possessions, purchases, musical preferences, websites visited, words used in Facebook posts, and “likes” registered on social media, can be used to make inferences about their unobservable inner world (Rentfrow and Gosling, 2003; Golbeck et al., 2011; Kosinski et al., 2013; Wang and Kosinski, 2018). For example, deep learning neural networks are now better at detecting people's sexuality from their faces than other humans are, posing a “threat to the privacy and safety of gay men and women” (Wang and Kosinski, 2018, p. 246). Social network users have been shocked by reports that detailed personal information about them could be predicted from simply what pages on Facebook they had “liked” (Kosinski et al., 2013).

This is utilized for financial and political ends. Surveillance capitalism claims human experience as free raw material that can be used to deduce “thoughts, feelings, intentions, and interests,” predict behavior, and then be monetized by selling such predictions to advertisers (Zuboff, 2019, p. 81). Micro-targeting uses personality data gathered on individuals to target them with political adverts which should, in theory, be maximally effective. This was at the heart of the Cambridge Analytica scandal (Cadwalladr, 2018). Whilst the effect of micro-targeting in changing political views is probably small (Hersh and Schaffner, 2013; Liberini et al., 2018; see also Kalla and Broockman, 2018), it is likely to have a greater effect on turnout (Bond et al., 2012). In close political contests, this could have decisive effects.

Whilst it is always difficult to separate political bluster and marketing spiels from fact, the use of behavior-reading poses a potential threat to the FoT of technology users. A century ago, U.S. Supreme Court Justice Brandeis crusaded against big banks use of “other people's money” (Rosen, 2016). Today, we need to address big tech's use of other people's data.

### Brain-Reading

Whilst care needs to be taken to avoid overstating the ability of neuroscience to decode thoughts from people's brain activity (“brain-reading”), there has been significant progress in this area. Whilst some work has studied how political stances can be predicted from brain structure (Kanai et al., 2011) and function (Schreiber et al., 2013), the majority of research has focused on decoding specific thoughts and perceptions.

Early studies were able to predict with a high degree of accuracy what type of pre-defined object (face, house, cat, etc.) an individual was looking at, based on their neural activity (Haxby et al., 2001). Technology can now use people's neural activity to predict what novel images people are viewing (Kay et al., 2008; Naselaris et al., 2009). Neuroimaging could once only create rudimental reconstructions of what an individual was seeing (Miyawaki et al., 2008). Now it can create “remarkable” reconstructions (Nishimoto et al., 2011). Brain imaging data can also be used to decode what people are hearing (Mirkovic et al., 2015) and predict what objects people have dreamt of (Horikawa et al., 2013).

Research has also attempted to infer individuals' verbal thoughts from their neural (Martin et al., 2014; Wang et al., 2017) and throat musculature activity (Jorgensen et al., 2003) with some success. Neural activity when people mime speech can now be directly converted into recognizable speech (Anumanchipalli et al., 2019). A major advance has been the ability to decode thoughts individuals are having which the decoding system has not previously been trained to recognize (Anderson et al., 2016; Pereira et al., 2018). Such approaches work on the principle that words are represented in the brain as semantic vectors. That is, any word can be represented by the extent it is associated with a finite number of specific features (Anderson et al., 2016). Each word in a basic 30,000 word vocabulary can be represented by a unique pattern of scores on these vectors. Once you know the neural activity associated with each of these features, you can potentially decode any word someone thinks.

Basic intentions can be decoded too (Haynes et al., 2007). It is even possible to predict what participants will do (for very basic tasks) before they themselves know (Soon et al., 2008). The ability to use this information to predict people's intentions in real-time is still limited. For example, the ability to predict people's intentions from their brain activity whilst they are playing a first-person shooter computer game in a scanner is currently limited to predicting intended movements. Intentions to fire cannot be detected due to the neural signal being swamped by a surge of activation associated with the emotions of firing or being fired upon (Smith, 2013).

Much of this work is preliminary, with accurate predictions only being possible under highly constrained experimental conditions. Yet funding is pouring in. Governmental funding in the United States has come from the Army, Air Force, Intelligence Advanced Research Projects Activity (IARPA) and Defense Advanced Research Projects Agency (DARPA) (Martin et al., 2014; Wang et al., 2017; Yuste et al., 2017), suggesting national security interests in brain-reading. Significant private investment is also occurring. Annual spending on neurotechnology by for-profit industry is estimated to be US$100 million per year and growing (Yuste et al., 2017). Facebook has announced plans for a Brain Computer Interface that aims to decode users' thoughts and transmit them to Facebook (Solon, 2017), and is actively funding research in this area (Anumanchipalli et al., 2019). Microsoft has patented brain-reading technology (Keskin et al., 2018). Elon Musk's start-up, Neuralink, is also trying to develop brain-computer interfaces (Marsh, 2018). Progress could be rapid and have profound implications for FoT.

### Taking Action Early: The Precautionary Principle

The law has long sensed the potential for technological advances to threaten FoT. “Advances in the psychic and related sciences,” prophesized U.S. Supreme Court Justice Brandeis in 1928, “may bring means of exploring unexpressed beliefs, thoughts and emotions” (*Olmstead v. United States*, 1928). In the 1970s, U.S. Supreme Court Justice Brennan noted that, “[t]he central storage and easy accessibility of computerized data vastly increase the potential for abuse of that information, and I am not prepared to say that future developments will not demonstrate the necessity of some curb on such technology” (*Whalen v. Roe*, 1977).

Such threats have now either materialized or are imminent (Hanson et al., 2019). The Morningside Group (a collaboration of neuroscientists, neuro-technologists, clinicians, ethicists, and machine-intelligence engineers) recently observed that “technological developments mean that we are on a path to a world in which it will be possible to decode people's mental processes and directly manipulate the brain mechanisms underlying their intentions, emotions and decisions” (Yuste et al., 2017). As such, they argue that it is “crucial to consider the possible ramifications now” (p. 160). Considering the implications of such technological developments for the right to FoT is one such crucial task.

A clear justification for this is offered by the precautionary principle. The World Commission on the Ethics of Scientific Knowledge and Technology (COMEST) defines the precautionary principle as: “When human activities may lead to morally unacceptable harm that is scientifically plausible but uncertain, actions shall be taken to avoid or diminish that harm.” (COMEST, 2005, p. 14). It defines actions as “interventions that are undertaken before harm occurs that seek to avoid or diminish the harm.” As Alegre (2017) has argued, technological developments that have the capacity to interfere with our FoT fall “clearly within the scope of ‘morally unacceptable harm”' (p. 230).

## Challenge II: New Understandings of Thought in the Twenty-First Century

### What Is Thought?

One problem for developing the right to FoT is that no definition of “thought” has been provided. As Loucaides (2012) notes, “there is no adequate material in the preparatory works of the drafters of the European Convention regarding the concept of ‘thought”' (p. 80). When studying the mind, psychologists tend not to use the blanket concept of “thought.” They work with more clearly specified mental processes, including memory, attention, inner speech, mental imagery, decision making, and planning (Alderson-Day and Fernyhough, 2015). Philosophical investigations of mental autonomy mirror this (Metzinger, 2013). As the purpose of FoT is to facilitate mentally autonomous individuals, discussions of the right to FoT need to track this approach.

It will hence be important to identify the key elements of thought that enable mental autonomy. Metzinger (2013) has proposed these are attentional agency and cognitive agency. Attentional agency is the ability to control one's focus of attention. This ability is under increasing threat as social media uses insights from the behavioral sciences to design products that more and more effectively hijack and retain attention (Eyal, 2014; Pandey, 2017), to the point of addiction (Kuss and Griffiths, 2017). The negative effects of this can be specific. The hijacking of parent's attention by smartphones can have detrimental effects on their families (Kildare and Middlemiss, 2017). However, there is also a wider problem; the person who cannot control their attention cannot control their thoughts. And, as Metzinger (2015) notes, “for as long as one cannot control one's own thought one cannot count as a rational individual” (p. 272).

Attentional agency is needed for cognitive agency, the ability to control goal/task-related, deliberate thought (Metzinger, 2013). An essential element of cognitive agency is the second-order mental action. To understand what this is, it is necessary to consider “hierarchical” accounts of autonomy (e.g., Frankfurt, 1971). These begin with the thoughts, desires and impulses that well up within us, termed “first-order” mental actions. To act on these, without reflection, is to fail display autonomy. Frankfurt (1971) called creatures who only acted in this way “wanton.” In his view, they were “not persons” (p. 11). An alternative response to first-order mental actions is to perform more mental work (a “second-order” mental action) to determine if such thoughts/desires/impulses are authentic, i.e., consistent with one's own chosen values and goals. Second-order mental actions make thoughts and desires “more truly one's own,” and allow people to recognize that their first-order thoughts may represent “a force other than” their own (Frankfurt, 1971, p. 13). They allow us to structure our thoughts, undertake logical trains of thought, and guide our behavior (Metzinger, 2013). The ability to perform second-order mental actions should hence form a key target for protection by the right to FoT.

Another issue relevant to developing the right to FoT is a move away from the conception that thought is only “in the head.” Clark and Chalmers (1998) make a persuasive case that the border between mind and world is not always constituted by the skin and skull. They propose that mind extends into the world. For them, the use of pen-and-paper to perform calculations or the re-arrangement of Scrabble tiles to make words, constitutes thinking. Clark and Chalmers argue that if “a part of the world functions as a process which, were it done in the head, we would have no hesitation in recognizing as part of the cognitive process, then that part of the world is (so we claim) part of the cognitive process” (p. 8). For example, in someone with dementia, a notebook could be functionally equivalent to memory.

In order for X to be classified as part of the extended mind, Clark and Chalmers (1998) propose three criteria: (1) X is a constant in the person's life and they will rarely take action without consulting it; (2) the information from X is directly available without difficulty; and, (3) upon retrieving information from X it is automatically endorsed. In 1998 Clark and Chalmers argued internet use was likely to fail to meet these criteria. Yet today, a case can be made that, when questions arise, much of the population will typically consult a search engine (e.g., Google), which they will always have access to without difficulty (via smartphones), which will lead them to trusted sources they will endorse. Internet searches have become thought.

The right to FoT should protect thought wherever it is found, not just in the head. This is important because if external thinking (e.g., internet searches, diaries, notebooks) is not deemed thought then it will only be protected by rights that can be permissibly violated under certain circumstances (e.g., the right to privacy). If it is deemed thought, then it will be protected by the absolute right of FoT. Any court that recognized the existence of external thought would be setting a highly impactful precedent.

### Advances in Understanding Decision-Making

The extensive literature on factors that affect human decision making (Cialdini, 2013) includes the suggestion of two distinctly different systems that we possess for making decisions (Kahneman, 2011). System 1 (“rule-of-thumb” thought) is evolutionarily ancient, shared with animals, effortless and allows quick judgments to be made by using rules of thumb called heuristics. Its speed comes at the cost of accuracy. In contrast, system 2 (“rule of reason” thought) is a slow, consciously controlled process of reasoning, which is evolutionarily new, unique to humans, and linked to language. Its cost (slower decision making) can be offset by its increased accuracy. Attempts by governments and corporations to encourage citizens to use rule-of-thumb thinking, bypassing rule-of-reason thinking, could potentially violate the right to FoT.

Knowledge of what influences decision making is already being used by the state to influence behaviors. Governments “nudge” citizens into joining organ donor registers, increasing fruit and vegetable consumption, and increasing tax collection rates (Thaler and Sunstein, 2008; Marteau et al., 2011). Such “libertarian paternalism” is argued to still permit choice (and hence be ethical), even if it does bias individuals to make certain choices (Thaler and Sunstein, 2008). The commercial use of such knowledge to manipulate consumer's thoughts and behaviors is more ethically problematic (Ariely and Berns, 2010). Research into biases in human decision making have been utilized by the private sector to enhance the probability of consumers making purchases (Eyal, 2014). These techniques take advantage of foibles of the human mind, rather than engaging with rational faculties to encourage purchasing behavior. This arguably raises issues relating to FoT.

Social networking companies also make use of behavioral science insights. Their business models are based primarily on advertising income, motivating them to maximize the time people spend using their product. Sean Parker, the first president of Facebook, recently discussed the thinking that went into building this social network. He described it as being “All about how do we consume as much of your time and conscious attention as possible?” and doing this by “exploiting a vulnerability in human psychology… The inventors, creators, it's me, it's Mark [Zuckerberg]… understood this consciously. And we did it anyway” (Pandey, 2017). Similarly, Guillaume Chaslot, a computer programmer who worked with YouTube engineers on its recommendation system, describes how “Watch time was the priority… Everything else was considered a distraction” (Lewis, 2018). Social networking sites achieve this by methods including variable reward reinforcement schedules (Ferster and Skinner, 1957), deployed in the context of providing information relevant to our fundamental need to belong (Baumeister and Leary, 1995) and desire for social status (Anderson et al., 2015). The imbalance in power resulting from the behavioral science knowledge of designers, whether they are creating social networks or casinos (Schüll, 2014), and the users of these products, raises the concern that users' FoT may be being violated.

In summary, our concept of what thought is and how the mind works have important implications for our conception of what the right to FoT should be. Before turning to a further consideration of these issues, there is the need to examine how the right to FoT is currently conceptualized.

## The Right to Freedom of Thought

The right to FoT formally became international law in 1976 as part of Article 18 of the International Covenant on Civil and Political Rights (ICCPR). Today, it is a staple of most international human rights treaties. In Europe it is found in Article 9 of the European Convention on Human Rights (ECHR). In the United States, whilst elements of the Fourth and Fifth Amendment pertain to FoT, it is primarily the First Amendment that is understood to protect an individual's thought from Government interference (Richards, 2015). Although the First Amendment does not mention FoT explicitly, U.S. courts have noted that a concern for FoT was present at the time of its adoption and that it is closely associated with it (Kolber, 2016). U.S. courts have explicitly referred to a “First Amendment right to freedom of thought” (*Doe v. City of Lafayette, Indiana*, 2003) and the U.S. Supreme Court has stated that “at the heart of the First Amendment is the notion that an individual should be free to believe as he will” (*Abood v. Detroit Board of Education*, 1977).

Reflecting the fundamental importance of the right to FoT, both the ICCPR and ECHR give it the status of an absolute right. This means it cannot be interfered with in any circumstances. Article 4(2) of the ICCPR sets out the “fundamental character” of this right, which is “reflected in the fact that this provision cannot be derogated from, even in time of public emergency.” In the United States, the right to FoT is as close to an absolute right as any that exists in the Constitution (Blitz, 2010; Richards, 2015). However, whilst one view of the First Amendment is that it protects thought itself, another view is that it only protects thought when intertwined with expression (Kolber, 2016). Furthermore, U.S. courts have made statements that question whether the First Amendment can be seen as offering absolute protection (e.g., *Branti v. Finkel*, 1980). For example, *Doe v. City of Lafayette, Indiana* (2003), saw reference being made to a potential “exercise of determining what state interests might outweigh a person's right to think.”

The profound importance given to this right by the law contrasts with an almost complete lack of law clarifying what it actually entails. The United Nations Human Rights Committee (UNHRC) have stated that the scope of the right to FoT is “far-reaching and profound; it encompasses freedom of thoughts *on all matters*” (United Nations Human Rights Committee, 1993, italics added). Yet the right appears to be so profound as to deter any attempts at actually defining it. As Bublitz (2011) notes, “In its over 50 years of existence the ECHR has only decided a handful of cases regarding freedom of thought…[it] is an almost empty declaration. There are no definitions over its meaning, scope or possible violations” (p. 103). Similarly, the U.S. Supreme Court has never stated exactly what the right to FoT is (Blitz, 2010).

This lack of development appears to be because the mind has not traditionally been conceived as an entity vulnerable to external intrusions or interference and has hence not been viewed as in need of legal protection (Bublitz and Merkel, 2014; Richards, 2015). This view can be seen in 1942 when the U.S. Supreme Court declared that: “[F]reedom to think is absolute of its own nature, the most tyrannical government is powerless to control the inward workings of the mind.” When the Universal Declaration on Human Rights was drafted a few years later, delegates remarked: “It would be unnecessary to proclaim freedom of [the inner sphere] if it were never to be given an outward expression” as the inner is beyond any access (Hammer, as cited in Bublitz and Merkel, 2014).

A useful way to frame an exploration of the limited case law and scholarly writing on FoT is through Vermeulen's (2006) non-binding commentary on Article 9 of the ECHR. Vermeulen proposes the right to FoT has three key elements; a right not to reveal one's thoughts, a right not to have one's thoughts manipulated, and a right not to be penalized for one's thoughts. I will utilize this framework, exploring, for each element, descriptive aspects (what does law say about this right), normative aspects (why should this right exist), and contemporary challenges that will need to be taken into account when developing the right.

## The Right not to Reveal One's Thoughts

### Descriptive Aspects: Law as It Stands

In the United States the concept of mental privacy developed in the late nineteenth century from laws surrounding physical privacy. Warren and Brandeis (1890) argued for a right ‘to be let alone', which included protection for “thoughts, sentiments and emotions” (p. 205). Since this time U.S. courts have clarified that an individual's right to privacy (guaranteed by the Fourth Amendment) includes mental privacy. In *Long Beach City Employees Assn. v. City of Long Beach* (1986), California's Supreme Court stated that “[i]f there is a quintessential zone of human privacy it is the mind.” More generally, the U.S. Supreme Court has stated that “The Constitution protects the right… to be generally free from governmental intrusions into one's privacy and control of one's thoughts” (*Stanley v. Georgia*, 1969).

In Europe, Article 8 of the ECHR (“Right to respect for private and family life”) protects a space where one can be free from unwanted intrusions, whether that “be that the head or the home” (Marshall, 2009, as cited in Bublitz, 2013, p. 14). Mental privacy can also be understood as being protected by Article 9 of the ECHR (“Freedom of thought, conscience and religion”). Case law is sometimes unclear which of these Articles violations of mental privacy should be understood as involving (e.g., *Folgerø Others v. Norway*, 2007). Yet, it is important to be clear which ECHR Articles guarantee mental privacy. If given by the right to FoT, it will be an absolute right. If given by the right to privacy, it would have to be balanced against other concerns.

### Normative Aspects: Why Should This Right Exist?

Mental privacy can be defined as our ability to determine for ourselves when, how, and to what extent information about our thoughts is communicated to others (cf. Westin, 1967). It reflects the idea there is a (contextually dependent) boundary between ourselves and others that must be respected (Richards, 2015). One argument for mental privacy is that it serves a protective function, defending us from distress at each other's less laudable thoughts and inclinations (Nagel, 1998). Another is that it facilitates intimacy, allowing us to choose to only bring certain individuals into our inner world, thereby acting as a marker of esteem or love (Nagel, 1998). However, the strongest argument for a right to mental privacy is that without it our mental autonomy would be diminished. “[A] lock on the door” noted Virginia Woolf (2001) “means the power to think for oneself” (p. 125). This is because exposure of our thoughts would effectively alter them by pressurizing us not to think certain things. As Cohen (2000) puts it, “Examination chills experimentation with the unorthodox, the unpopular, and the merely unfinished” (p. 1426).

The argument that mental privacy is necessary for mental autonomy rests on two claims. The first is that if we cannot keep our thoughts private then we will experience conformity pressures to think in a certain way. The second is that this pressure will cause self-censorship, impairing mental autonomy. What evidence is there for these claims?

We are clearly motivated to make our observable behaviors conform to social norms. Normative conformity (adjusting one's behavior in order to fit in with the majority) has been extensively documented and appears to exist because it serves three purposes (Cialdini and Goldstein, 2004). It generally enhances accuracy of decision making, enhances social approval leading to greater access to resources, and helps enhance, repair, and protect self-esteem (Cialdini and Goldstein, 2004). Conformity hence offers significant survival benefits.

As a result, our brains have evolved to motivate us to conform. The brain recognizes when we are deviating from social norms and reduces activation in areas associated with reward (e.g., the nucleus accumbens; Klucharev et al., 2009). Failing to conform is associated with increased activity in the amygdala, the brain's fear center (Berns et al., 2005), and in a number of other neural markers of stressful and aversive experiences (Yu and Sun, 2013). Thus, if our thoughts were made (or threatened to be made) public against our will, we would experience biologically rooted pressures to conform them to social norms.

Would such pressures lead our thoughts to conform? It is well-established that conformity pressures often result in people conforming their speech and behavior to group norms (Asch, 1952). Yet, behaviors are something we can reflect upon and potentially change. As we do not consciously choose our first-order thoughts, how could we make them conform to a standard?

Mental conformity could firstly occur by a conscious process of inhibiting thoughts as they arise. People use a range of techniques to control such thoughts, including punishment, worry, distraction, social control, and reappraisal (Wells and Davies, 1994). Yet mental conformity could also occur by non-conscious mechanisms. For example, Eriksen and Kuethe (1956) presented participants with a list of 15 words, and asked them to free associate other words related to these. Five of the words participants generated were followed by electric shocks. They were then asked to free associate with the same words again, but this time with no shock. It was found that participants showed a decrease in responding with the words that were punished by electric shock earlier. Some were not aware that the shocks were why they were doing this. Mental conformity can hence occur through an insidious, non-conscious process of self-censorship.

Evidence of self-censorship in a democracy as a result of violation of mental privacy can already be found if (as argued above) we accept that internet searches represent thought. A chilling effect on individual's internet search behavior was found to result from the June 2013 revelations of large-scale internet surveillance of individuals, as part of the information divulged by Edward Snowden. In this study, Marthews and Tucker (2017) collected data on internet search term volume before and after June 2013. They found that after this date there was a 10% drop in search terms that were assessed as potentially getting individuals in trouble with the government, as well as a significant drop in use of terms that could be personally embarrassing if revealed.

There is hence a fundamental need for what Richards (2015) has termed intellectual privacy, defined as a “zone of protection that guards our ability to make up our minds freely… the protection from surveillance or unwanted interference by others when we are engaged in the process of generating ideas” (p. 96). This should form an important pillar of the right to FoT.

Arguments against mental privacy are centered on benefits which pale by comparison to what would be lost if this right vanished. Richmond (2012) notes a potential benefit to making minds more transparent is the normalization of abhorrent first-order thoughts, reducing self-stigma and shame. Yet, this could equally well be done by other less intrusive means, such as public education (McCarthy-Jones, 2019). Opponents of data privacy argue that the more information that marketers have, the better they can serve consumers and the less intrusive they need to be (Cohen, 2000). Both law and economics view one party having more knowledge than another as being inefficient as it hinders fully informed exchanges (Cohen, 2000). Privacy, by acting as a barrier to prediction (which is central to surveillance capitalism), becomes a barrier to efficiency. Yet, “a relentless focus on efficiency is the surest way to destroy liberty” (Rosen, 2016, p. 77). Furthermore, this case against mental privacy is an economic one, not a moral one. As Ryssdal and Garrova (2017) put it “The reason we don't have any privacy is because people can make money off of our not privacy.” It is not because it is in the interest of individuals to have no privacy. Indeed, without privacy we would, to a meaningful extent, not exist at all.

### Contemporary Challenges That Should Shape the Right Going Forward

We have a natural ability to work out, to some degree, what others are thinking. This is termed “theory of mind” (ToM). In a typically developing child the basic form of ToM develops at around four-years-old (Wellman et al., 2001). The evolution of ToM, which only exists in its full blown form in humans, was presumably driven by the benefits of being able to predict another's behavior (Shultz and Dunbar, 2012). As humans evolved the ability to work out others were thinking, this created selection pressures for the ability to conceal one's thoughts from another. This in turn put selection pressures on the ability to detect deception. It turns out that our ability to lie is significantly better than our ability to detect lying. Adults can only detect lies told by children at a rate slightly better than chance (Gongola et al., 2017). Even as children we have a powerful ability to encrypt our inner world. However, the development of brain- and behavior-reading techniques threaten to disturb the delicate balance between our evolved ability to know other's thoughts yet to also shield our inner world. But what exactly, as Ryberg (2017) has asked, “constitutes the difference between these two ways [natural and technological] of approaching other people's mental life”?

One answer is that the inappropriateness of brain- and behavior-reading stems from its supra-natural ability to infer a person's thoughts, steamrollering through evolved defenses, creating a significant power imbalance between the possessors of such technology and their “targets.” Power imbalances between the sender and recipient of a message has previously been ruled by the European Court of Human Rights to create an improper influence on thought (*Larissis and Others v. Greece*, 1998). The possessors of brain- and/or behavior-reading technology could hence be deemed to have “improper access” to the minds of other individuals. In addition, internet-use is so ubiquitous today that individuals may feel they have no choice but to surrender their data for behavior-reading, in exchange for services. This suggests a coercive element.

Another way to argue that brain- and behavior-reading is inappropriate, from a European perspective, is to start from the right to informational privacy given by EU Regulation 2016/679 (General Data Processing Regulation: GDPR). GDPR has no problems with one person naturally working out what another is thinking from their behavior (“data”) in everyday life. This would be legitimately processing someone's data as “a natural person in the course of a purely personal or household activity” [EU Regulation 2016/679, Art. 2, 2(c)]. However, if a company starts processing someone's data for the purposes of brain- or behavior-reading then they would need their explicit consent to do this. If they do not ask, or the person does not grant such consent, then such processing would be illegal. Of course, the GDPR has many permissible violations (e.g., national security). If the right to FoT covered such invasions of mental privacy, no violations would be permissible.

This raises the question as to whether the right to mental privacy should be absolute. It is possible that society may wish permissible violations to exist. Warrants can be sought and granted to search individuals' homes. It is not immediately obvious that there should not be similar permissible violations for the mind. It is possible to imagine a judge granting a warrant for a mental search, which could take the form of an algorithmic analysis of all an individual's available data and/or the collection of neuroimaging data. The prosecution of hate crimes already often involve ransacking a person's life in order to determine what they were thinking (Corry, 2000).

Part of a justification for permissible violations could be the claim that the right to mental privacy needs to be balanced against other interests and rights. For example, Bublitz (2014) offers a thought experiment of a future in which outward signs of particular mental states (i.e., behavior-reading) could be observed to uncover individuals' thoughts in sensitive places such as banks, airports or even public parks. This would aim to deter the occurrence of such thoughts, leading to what Bublitz terms “zones of restricted freedom of thought” (p. 15). It is easy to see how this could slide into a dystopia, with people being punished for thoughts deemed dangerous. This highlights that most permissible violations of mental privacy would only make sense if people could justifiably be punished for such thoughts. But can they?

## The Right not to be Penalized for One's Thoughts

### Descriptive Aspects: The Law as It Stands

“[I]f there is any one proposition that commands general agreement among theorists and practitioners of the penal law,” Brudner (2009) has argued, “it is that judicial punishment ought not to be inflicted for private thoughts” (p. 108). Yet, in one sense it is common to punish thoughts. The famous maxim of criminal law states that an act does not make a defendant guilty without a guilty mind. In this sense, every crime with a *mens rea* requirement technically involves the person being punished for thoughts. Similarly, in the case of hate crime, Corry (2000) has argued that “[b]ecause these laws seek to punish certain unacceptable prejudiced thoughts, more accurate terms for these laws are ‘thought crimes”' (p. 469).

Yet, it would not be correct to say people are being punished for “mere thought” in such cases. As was noted in *U.S. v. Gamache* (1998), “[p]roof of intent naturally means proving state of mind, but that does not mean that one is punishing ‘mere thought' any more than that the requirement of proving *mens rea* in most crimes means that one is solely punishing ‘mere thought.”' Some substantial step has to have been taken toward a criminal act, in addition to the thought, comprising “objective acts [which]…mark the defendant's conduct as criminal in nature,” meaning that a “fragment of the crime” is in progress (*U.S. v. Price*, 1998). U.S. courts have stated that the fear “that thoughts alone may encourage action—is not enough to curb protected thinking” (*Doe v. City of Lafayette, Indiana*, 2003). In the absence of any substantial steps toward a criminal act, law in the United States recognizes “the fundamental constitutional principle that a person's thoughts are his own—however distasteful they may be to the state or to the populace” (*Steffan v. Perry*, 1994). Mere thought is hence protected.

Yet, in prosecutions for attempted crimes it is possible to conceive that the person is being punished for a thought; namely, an intention (Austin, 1885). The substantial steps requirement only means that stronger evidence has been gained of the strength of the intention, it does not mean that something other than the intention is being punished (Morris, 1965). Yet, it can be argued that what the person is being punished for is conduct accompanied by a certain state of mind, which makes it illegal conduct.

A series of cases involving *Doe v. City of Lafayette, Indiana* (2001, 2003, 2004) are central to present concerns because they gave rise to “a set of facts that tests just how far the judiciary is willing to stretch and extend the unenumerated constitutional right of freedom of thought when the thoughts in question relate to committing one of the most heinous of crimes—child molestation” (Calvert, 2005, p. 138). Doe, a convicted sex offender, was driving home from work when he began to have sexual thoughts about children. He drove to a city park, watched children, had thoughts about having sexual contact with them, and then left without having any such contact. An anonymous source (potentially from his Sexual Addicts Anonymous group; Calvert, 2005) later reported Doe's thoughts to officials. The City decided, although Doe was no longer serving a sentence or on probation, to ban him from entering any City parks.

Doe challenged the ban, which a district judge upheld (*Doe v. City of Lafayette, Indiana*, 2001). Doe then appealed this decision to a three judge panel of the U.S. Seventh Circuit Court of Appeals (*Doe v. City of Lafayette, Indiana*, 2003). The Court concluded that Doe's actions did not “reach a level of criminal culpability necessary to justify punishment” and that his simple presence in the park, thinking sexual thoughts about children, did not qualify as a “substantial step” toward an attempted sex offense. They hence decided that the ban violated “Doe's First Amendment right to freedom of thought” by punishing him for “pure thought,” and overturned it. Eleven judges of the Seventh Circuit Court of Appeals then reheard the case (*Doe v. City of Lafayette, Indiana*, 2004). This time the court upheld the ban, reasoning Doe was banned from parks because of his actions, not because of his thoughts. Whether or not thought can be punished hence appears to be somewhat open to interpretation.

### Normative Aspects: Why Should This Right Exist?

In the nineteenth century, Sir William Blackstone (1832) stated that “as no temporal tribunal can search the heart, or fathom the intentions of the mind, otherwise than as they are demonstrated by outward actions, it therefore cannot punish for what it cannot know” (p. 14). As technological advances make the reliable assessment of inner states increasingly feasible, arguments for not punishing thought now need to be solidly based on principle.

The most influential, principle-based argument against punishing thought comes from the following syllogism. Major premise: We can only punish that which violates important interests of others in which they have a right. Minor premise: Thoughts cannot violate important interests of others in which they have a right. Conclusion: We cannot not punish thoughts. The major premise is termed the “harm principle.” This stems from John Stuart Mill's argument that the “only purpose for which power can be rightfully exercised over any member of a civilized community, against his will, is to prevent harm to others” (Mill, 1859/1985, p. 68).

The ex-post version of the harm principle states that coercion is justified only if it actually prevents or reduces harm, but in its ex-ante version coercion is justified if it prevents or reduces risk (Holtug, 2002). The difference is important. The question of whether a thought actually causes harm to others is very different to whether a thought increases the risk of harm to others. If reckless driving can be prosecuted as it increases risk that the law seeks to diminish (Feinberg, 1987), why could we not prosecute reckless thinking? It turns out that thought's importance to society, rather than its innocuousness, saves it.

The contention that thought cannot risk others is something of a strawman. First, it would be contradictory to say that we need FoT to be able to control our behavior, but that our thoughts can't risk anyone. Second, a core tenet of contemporary evidence-based psychology is that our thoughts can harm us and others through influencing our behaviors, emotions and physiology (Beck and Haigh, 2014). Third, if overt speech can incite others to act and thereby cause harm, then it logically follows that our covert, inner speech can incite ourselves to act and thereby risk others. The Russian psychologist Lev Vygotsky (1978) was clear that a central purpose of our inner speech is to allow children to self-regulate their behavior and thoughts. As he put it, the use of verbal mediation means that humans are able to “control their behavior from the outside” (p. 40). To completely decontextualize thought from action is to fundamentally misunderstand its purpose.

Better questions are whether different thoughts are associated with different levels of risk, which have most risk, and how great this risk is. It can be argued that risk of harm from thoughts is typically low. The psychological research literature is replete with examples of people's beliefs being poor predictors of their behavior (e.g., LaPiere, 1934). Whilst some have expressed extreme pessimism at the ability of attitudes to predict behaviors (Wicker, 1969), the research literature on this topic is complex (Kruglanski et al., 2015). Nevertheless, it is generally the case the attitudes predict behavior to a lesser degree than we might expect.

Yet some categories of thoughts clearly pose more risk of harm than others. The theory of planned behavior argues that behavior can be best predicted from intention (Ajzen, 1991). Indeed, as Mendlow (2018) observes “There would be little point to forming intentions if intentions didn't generally increase the likelihood of actions.” (p. 2017). Not only can intentions create risk of harm but, as Mendlow observes, in the case of an intention to kill formed after extensive reflection and deliberation they can arguably be associated with a comparable risk of harm to actions that we already criminalize on account of their dangerousness, such as driving recklessly or possessing volatile explosives. Duff (2007) also suggests a potentially harmful category of thoughts are those for which the completion requires overt action, with intention formation being an example of this.

Another type of thought that increase risk of harm are second-order thoughts that endorse and encourage unwanted, unacceptable first-order thoughts. Someone who fails to find unwanted, unacceptable first-order thoughts repugnant, who does not try to suppress or avoid them, who does not try to avoid situations that trigger them, or is aroused or acts on them, may lead us to have cause for concern (Veale et al., 2009). Someone who felt this way about intrusive sexual thoughts they had about children is a potential sexual offender (Veale et al., 2009). This can be contrasted with Doe's claimed second-order thoughts in *Doe v. City of Lafayette, Indiana* (2004), which rejected his first-order sexual thoughts (“I was not planning to act on my thoughts. I recognized that these were just unhealthy thoughts and I realized I needed to leave the park, which is what I did”).

Even though thoughts can increase risk of harm, to punish them it would still need to be shown that the thinker merited condemnation or blame, i.e., was culpable for them (Mendlow, 2018). In the case of an intention to kill, reached after extensive deliberation (second-order thought), condemnation and blame are clearly deserved (Mendlow, 2018). Yet, we are not culpable for all our potentially harmful thoughts. Unwanted, intrusive and abhorrent first-order thoughts are common (Purdon and Clark, 1993; Byers et al., 1998) but one should not be deemed culpable for them (McCarthy-Jones, 2019).

Having demonstrated that some thoughts can be both dangerous and culpable, a remaining objection to punishing thought stems from the gap between intent and action. Punishing at the stage of mere intent diminishes the autonomy of the person by not allowing them to self-govern and restrain themselves from performing the act. Waiting for significant preparatory steps to be taken gives people the maximum autonomy to change their mind, before the law steps in coercively (Morris, 1965). However, Mendlow (2018) notes that such arguments assume that someone “hasn't already committed a punishable wrong *simply by preparing or intending*.” (p. 2018, italics in the original). He also notes that “To intervene on the expectation that he'll follow through on his criminal choice is arguably to show *respect* for his capacity for self-government.” (p. 2365). However, a practical reason not to punish intentions comes from Morris (1965). He notes that if people were to be punished simply for their intentions then, once their intention had formed, they would have no incentive not to perform the act. Not punishing mere intentions rewards self-restraint.

All this brings us to the point where we now need to explain, as Mendlow (2018) puts it, “why it's intrinsically unjust to punish mental states that are provable, dangerous, and culpably wrongful: mental states that bear the chief hallmarks of paradigmatic punishable actions (p. 2360). Mendlow's answer is complicated, and the reader is referred to his excellent treatment of this question. His basic idea is that punishment for mere mental states is intrinsically unjust because it represents, indirectly, mind control, and “it's *because* the state mustn't control thoughts that the state mustn't punish them” (p. 2018).

Another argument against punishing thought stems from a consideration of what is required for related rights, such as the right to freedom of speech, to be limited. Article 19 of the ICCPR states that limitations may be placed on freedom of expression in certain cases and if one can “demonstrate in specific and individualized fashion the precise nature of the threat, and the necessity and proportionality of the specific action taken, in particular by establishing a direct and immediate connection between the expression and the threat” (United Nations Human Rights Committee, 2011). In the United States, the Brandenburg test stipulates that speech can only be limited if it is “directed to inciting or producing imminent lawless action and is likely to incite or produce such action” (*Brandenburg v. Ohio*, 1969). It is likely to be hard to show an immediate/imminent connection between a thought and a threat.

Furthermore, when we consider how strong the substantial action test is for establishing “attempt,” in the case of observable behaviors in the United States, it is hard to see how mere thought could be deemed attempt. For example, as Duff (1998) notes, in *United States v. Still*, the defendant was found wearing a disguise whilst parked in a van 200 feet from a bank. After his arrest, the defendant said to the police “You did a good job. You caught me five minutes before I was going to rob a bank. That's what I was putting the wig on for.” And still the defendant was not convicted of attempt. If preparations this clear cannot be deemed attempt, it is hard to see how the thoughts of a defendant, on their own, could ever be deemed attempt and punished.

### Challenges That Should Shape the Right

In time, greater access to the thoughts of individuals, through brain- and behavior-reading, will raise the question of whether, once we can know such thoughts with an appropriate degree of certainty, we should punish them. If, despite the above arguments against punishing thoughts, the public and their elected representatives were to support punishment for thought, why might his be? One answer is that this would be being done “to calm the anxiety of others for their physical security or psychic integrity, or simply to achieve dominance for their moral opinions” (Brudner, 2009, p. 108).

Even without new legislation, judges' views on the punishment of thought may be influenced by a risk-adverse public clamoring for such punishment. As the former Justice of the Supreme Court of the United Kingdom, Lord Sumption (2019), has noted, “Judges don't decide cases in accordance with the state of public opinion but it is their duty to take account of the values of the society which they serve. Risk aversion has become one of the most powerful of those values and is a growing influence in the development of the law.”

## The Right not to Have One's Thoughts Manipulated

### Descriptive Aspects: The Law as It Stands

The right to not have one's thoughts manipulated is recognized as settled law in many countries. The U.S. Supreme Court has repeatedly ruled that it is unconstitutional for the government to interfere with people's thoughts. Some U.S. courts have viewed this as part of the constitutional right to privacy, which “is broad enough to include the right to protect one's mental processes from governmental interference” (*Rennie v. Klein*, 1981).

More often, courts have simply recognized that the state must not control the thoughts of its citizens. In *Stanley v. Georgia* (1969), the U.S. Supreme Court ruled that the defendant could not be forbidden to privately possess obscene material (a film of adults engaged in sex acts) because to do so would be for the government to claim the right to “control the moral content of a person's thoughts.” In its ruling the Court noted that “[o]ur whole constitutional heritage rebels at the thought of giving government the power to control men's minds.” In *Ashcroft v. Free Speech Coalition* (2002), the U.S. Supreme Court ruled that someone could not be punished for possessing visual depictions of an actor who appeared to be a minor engaging in actual or simulated sexual intercourse. Simulated child pornography was deemed not to involve direct harm to a child, and hence the Court decided that the only case that could be made to ban simulated child pornography was that it had a negative effect on a viewer's mind. Specifically, it would be being banned on the basis that it “whets the appetites of pedophiles and encourages them to engage in illegal conduct.” The Court deemed this an impermissible basis for criminalizing it, quoting *Stanley* in concluding that “The government ‘cannot constitutionally premise legislation on the desirability of controlling a person's private thoughts.”'

The 2009 Charter of Fundamental Rights of the European Union introduced the concept of a “right to mental integrity.” Article 3.1 of the Charter states that “Everyone has the right to respect for his or her physical and mental integrity.” It does not indicate what mental integrity means though, and leaves its interpretation to the courts (Bublitz, 2011). However, Mendlow (2018) has defined this as; “the right to be free from unwanted mental interference or manipulation of a direct and forcible sort.”

The right to FoT has been suggested to protect thought against manipulation. Nowak (1993) has argued that the right to FoT means “the right of everyone to develop autonomously thoughts… free from impermissible external influence” (p. 412). Nowak gives examples of impermissible external influences, namely the use by states parties of indoctrination, brainwashing, psychoactive drugs or other means of manipulation. He also claims influencing is impermissible “when it is performed by way of coercion, threat or some other prohibited means against the will of the person concerned or without at least his or her implicit approval” (p. 413).

Courts have also given some guidance on what they believe to characterize illegitimate forms of thought manipulation. One characteristic has been deemed to be the use of power imbalances to influence thought. In the case of *Larissis and Others v. Greece* (1998), the European Court of Human Rights concurred with the conviction of high-ranking officers who tried to convert lower-rank soldiers to Jehova's Witnesses. Due to the hierarchical structure of the army, the conduct of the officers could be deemed “improper” undue influence. The European Court of Human Rights has also suggested that improper means of influencing thoughts included exerting improper pressure on people in distress or need, violence, brainwashing, and unreasonable propaganda (*Kokkinakis v. Greece*, 1994). However, the Court offered no definition of “unreasonable propaganda.”

### Normative Aspects: Why Should This Right Exist?

Arguments against manipulating thought echo those from previous sections of this paper, namely the potential detrimental impact on mental autonomy.

### Challenges That Should Shape the Right

Better understandings of how the mind works create better methods for manipulating it. Not only have methods of manipulation changed but so has the medium in which manipulation occurs. “Minds,” as U.S. Supreme Court Justice Kennedy observed, “are not changed in streets and parks as they once were. To an increasing degree, the more significant interchanges of ideas and shaping of public consciousness occur in mass and electronic media” (*Denver Area Educational Telecommunications Consortium, Inc. v. Federal Communications Commission*, 1996). Today, there is particular concern about illegitimate influence through social media. For example, a controversial experiment run through Facebook explored the effects of manipulating the emotional content of people's newsfeeds on their mental state (Kramer et al., 2014). It found that increasing the number of negative stories increased the levels of negative emotions of users.

A key problem in this area is that there is no clear definition of what constitutes manipulation. One approach to this problem is to begin by defining what the key cognitive processes are involved in mental autonomy. As noted earlier, attentional and cognitive agency appear to be central to this ability (Metzinger, 2013). Circumvention of these could hence be defined as manipulation of thought. Another approach argues that the paradigmatic form of legitimately changing other's thoughts is rational argument (Bublitz, 2011; Bublitz and Merkel, 2014). In this view, rational argument cannot be said to violate FoT because “it expresses the spirit of the provision: free and uncensored exchange of ideas” (Bublitz, 2014, p. 21). In such an exchange, the better argument, wins by what Habermas termed ‘unforced force' (“zwanglose zwang”) (Bublitz, 2014). Illegitimate manipulation would then be characterized as the disabling or circumvention of an individual's ability to rationally appraise information. But what counts as circumvention? Could this include using insights from behavioral science to take advantage of system 1 rule-of-thumb thinking and the cognitive biases built into the human brain?

This issue is most salient when we consider marketing, which often tries to bypass rational self-control (Bublitz, 2014). As the psychologist John Bargh has noted, “methods to thwart or bypass the consumer's defenses against influence are becoming ever more powerful, and yet he [the consumer] remains as ignorant of these influences and … overconfident of his control” (Bargh, as cited in Bublitz, 2014, p. 13). Despite this, the regulation of marketing focusses on preventing deceptive or misleading information. The right to FoT suggests regulators should also be concerned by attempts to bypass rational self-control.

Many marketing tactics can be seen to encourage rule-of-thumb thought, thereby avoiding engagement of rule-of-reason thought. What is likely to be critical in determining whether this represents “manipulation” is whether such techniques diminish the *capacity* to reason or not. Arguably, they do not. Encouraging the use of rule-of-thumb thought, does not remove an individual's capacity to use rule-of-reason thought. Rather than limit the free speech of marketers, a less invasive way to deal with this issue would be to use public education (though this places the burden on the public, not the corporation). Simply knowing that others are trying to persuade us can increase mental autonomy. This is because a natural human reaction to others attempting to persuade us (Walster and Festinger, 1962) or limit our choice (Brehm, 1966) is to resist being persuaded. Naturally, marketing attempts to circumvent such resistance by limiting the salience of the attempt to influence. For marketers, the best way to prevent resistance is not to engender it in the first place.

This emphasizes the need for public education on the mechanisms of persuasion. This could include requiring advertisements to state what behavioral science insights they are using (e.g., loss/gain framing, social norms, etc.; Kahneman, 2011) in order to help consumers respond rationally with rule-of-reason thinking. Here, to paraphrase Brandeis, allowing sunlight to fall on the murky processes of manipulation may be the best form of disinfectant (Rosen, 2016). Yet even this may be ineffective. Knowing how social networking sites deploy techniques such as variable reinforcement schedules to get you hooked on their products does not automatically eliminate their pull, any more than knowing cigarettes use nicotine to make them addictive makes a smoker crave them any less.

Once a line has been drawn between legitimate influence and illegitimate manipulation, it can be asked if the right not to have one's thoughts illegally manipulated should be absolute. Mendlow (2018) notes that forcible manipulation of a person's mind is already sometimes permissible, such as in cases where the state is permitted to force certain prisoners with mental health difficulties to ingest psychiatric medication. The U.S. Supreme Court recognized this as lawful in *Washington v. Harper* (1990). Here the Court ruled that the justification for this entailed the person being “dangerous to himself or others” and that such treatment would be in their “medical interest.” In such cases, this intervention arguably looks to restore lost FoT, though this is a contentious area.

A likely problem with any attempt to regulate the “illegitimate manipulation” of thought is that such manipulation is likely to occur through activities deemed “speech,” which is also legally protected. The strong protection historically accorded to speech is likely to push the bar as to what counts as “illegitimate manipulation” very high. One case that saw something like such a balance being struck was *Kokkinakis v. Greece* (1994). In this case the European Court of Human Rights held that proselytism is an exercise of religious freedom protected by Article 9 of the ECHR (“Freedom of thought, conscience and religion”). However, the Court also held that the right of believers to remain free from unwanted conversion attempts was likewise protected by Article 9. As a result, both the missionary and the believer could appeal to this Article and a balance had to be struck. In this case, the court held that restrictions of proselytism are not necessary in a democratic society as long as conversion attempts do not involve illegitimate means to influence thought.

## Conclusions

### New Rights?

One approach to developing the right to FoT in the twenty-first century is to suggest that the novelty of the challenges faced necessitates new rights to address them. A need has been proposed for new rights to “mental self-determination” (Bublitz and Merkel, 2014), “cognitive liberty” (Boire, 2001), and “psychological continuity” (Ienca and Andorno, 2017). In contrast, Alegre (2017) has argued that these new proposed rights represent the practical development of the contours of FoT in the twenty-first century, and that there is no need to design new rights. Instead she proposes we need clearer guidance and legal development of the meaning of the right to FoT in the modern context and a more detailed legal framework to protect it.

I have argued that the law should develop the right to FoT with the clear understanding that what this aims to secure is mental autonomy. This process would begin by establishing the core mental processes that enable mental autonomy, such as attentional and cognitive agency. These should be placed at the center of the right to FoT. I also argued we should expand the domain of the right to FoT to cover external actions that are arguably constitutive of thought. This includes reading, writing, and many forms of internet search behavior. Such “thought” is currently not protected by the right to FoT and hence not sheltered by its absolute protection. This chills thought.

Mental autonomy can only be ensured by a prohibition on the illegitimate manipulation of thought. This in turn justifies the right to mental privacy and the right not to have one's thoughts punished, as both effectively manipulate thought. What society wishes to deem to be illegitimate manipulation is a substantive legal and public policy question, urgently needing debating. Here again, an understanding of the core mental processes underpinning mental agency (attentional and cognitive agency) is likely to be important in determining what should be deemed illegitimate manipulation.

The source of the threat also needs to be considered. Law must protect us from threats to FoT from both the state *and* corporations. The Founding Fathers of the United States saw the threats corporations could pose. As Jefferson put it “I hope that we shall crush in its birth the aristocracy of our monied corporations, which dare already to challenge our government to a trial of strength, and bid defiance to the laws of our country.” There is the need to educate the population on the threats to FoT posed by corporations (Chomsky, 1989). And it is not only the public that needs to be apprised of this threat. As Greenwood (2017) has noted, “the Supreme Court is quick to see the possibilities of governmental overreach, but much less willing to see the problems of ‘private,' let alone corporate, power.” (p. 221). It is time, Greenwood notes, to take corporations seriously “as our institutions, ultimately under our control” (p. 221).

Today, international human rights law treaties, such as the ICCPR, place direct obligations on states, but not corporations. States must ensure that they themselves do not violate human rights. However, they must also take steps to protect human rights from interference by non-state actors. This is the generally accepted interpretation of Article 2(1) of the ICCPR, which requires a state “to respect and to ensure to all individuals within its territory and subject to its jurisdiction the rights recognized in the present Covenant” (Knox, 2011). As the United Nations Human Rights Committee (2004) has clarified, a state will only have fully discharged its obligations under the ICCPR if “individuals are protected by the State, not just against violations of Covenant rights by its agents, but also against acts committed by private persons or entities.” Thus, corporations have an indirect duty not to violate the right to FoT, which should be enforced by states [ICCPR, Article 2(3)].

Whilst calls for human rights law to apply directly to corporations have been rejected (Knox, 2011), in 2011 the United Nations Human Rights Council endorsed the Guiding Principles on Business and Human Rights (United Nations, 2011). Guiding Principle 11 states that “Business enterprises should respect human rights. This means that they should avoid infringing on the human rights of others and should address adverse human rights impacts with which they are involved.”

States hence need to act to prevent corporations violating people's right to FoT, ensuring effective remedies where violations occur, and corporations need to ensure they respect the right to FoT, publically expressing this as a policy commitment and carrying out due diligence on how their activities may adversely impact the right to FoT. One may be skeptical as to whether corporations will actually do this unless new, enforceable laws, as opposed to Guiding Principles, require them to. In any case, customers voting with their feet may be a more efficient and effective solution. This raises the question of what ways there are to support FoT which do not require legislation.

One option is to employ techo-regulation, which involves the “the intentional influencing of individuals' behavior by building norms into technological devices” (van den Berg and Leenes, 2013, p. 68). The best known example of techno-regulation is the use of speed bumps, which by and through their design encourage drivers to stay within the speed limits set by a regulator (van den Berg, 2011). Whilst techno-regulation often functions so that people have no choice but to act in the desired regulatory pattern (van den Berg, 2011), to use it to *force* FoT would seem somewhat contradictory. Instead, the value of FoT could be embedded in the design of technology in an optional manner, such as by providing versions of technologies that support FoT, which people can freely choose to utilize.

One such approach could involve creating an on-line speed bump, which creates decisional friction. For example, the scraping of personal data by large tech firms in order to serve up personalized adverts or content suggestions (Zuboff, 2019) is helpful *if* we consciously decide that we want to act on this information. However, it can also be viewed as problematic if we consider it in relation to first- and second-order thoughts. When an advert or content suggestion “pops up” in front of us, chosen by the company based on our previous behavior, this can be seen to be equivalent to a first-order thought/desire that pops into our minds. Clicking on this unthinkingly, buying the product or advancing to the next YouTube video, means we have failed to use second-order thoughts to appraise whether we actually want this first-order thought/desire. We have not worked out if this is our own desire, or simply the desire of the corporation. Such a process encourages us to act in a way that we saw Frankfurt (1971) describe as “wanton” (p. 11). This may explain the feeling of disgust we can have after getting lost in products such as YouTube. Surveillance capitalism is encouraging a wanton world.

One solution to this would be to require corporations to provide information in an autonomy-supportive context. This would encourage choice and participation in decision-making, provide a rationale for why a particular decision is appropriate, minimize pressure and use non-pressuring language (Chatzisarantis et al., 2009). A feature of this environment would be a context that allows thought to be slowed down, making it more likely that responses will be governed by second-order, rule-of-reason thought. For example, users could have the option to create a moratorium of five minutes before they can respond on-line to a social media post (cf. Jacobs, 2018; Deibert, 2019), proceed to the next video, or finalize a purchase. Evidence that this promotes the use of reason comes from studies that show stronger arguments may only be more persuasive than weaker arguments when people are given sufficient time to think about them (Paxton et al., 2012).

Mental privacy could be facilitated by mandating search engines to provide an option of making users completely anonymous to facilitate free searching (thinking) or by enabling audience segmentation that allows users to maintain a range of identities (van den Berg and Leenes, 2011). Suggestions have also been made for the design of “humane tech” (Centre for Humane Technology, 2019a), and the use of apps to help users regain control (Centre for Humane Technology, 2019b), all of which can be seen to support FoT.

### Debating the Right to FoT

A core feature of democracy is that citizens contribute to the laws that bind them. Whilst it has been argued here that limitations on FoT are hard to imagine, and hence its status as an absolute right should stay in place, this is a matter for public debate. For example, if new technologies are deemed to threaten users' absolute right to FoT, this could stop the further development of such technologies. This may not be deemed desirable given the general benefits such technologies could have. Can a balance be struck here and, if so, how? One option, driven by the precautionary principle, would be to take what Mullender (2000) terms a qualified deontological stance. This would recognize FoT as intrinsically valuable, acknowledge that both it and a culture in which it can flourish are worthy of protection, and demand clear reasons for putting it at risk by brain- or behavior-reading. Another option, a curb-led approach of the form discussed by Brennan in *Whalen v. Roe* (1977), would take what Mullender (2000) terms a qualified consequentialist stance. This would prioritize the pursuit of generally beneficial outcomes from brain- or behavior-reading (e.g., security), whilst acknowledging that their potential detrimental impact on FoT means there must be limitations on how such practices are pursued, such that they do not act in ways that could be reasonably expected to violate FoT. Given the importance of the mental autonomy that FoT supports, a qualified deontological stance appears more appropriate. This is particularly the case given the tendency of government to subsume individual rights to the pursuit of the general interest (Mullender, 2000) and of corporations to subsume individual rights to the pursuit of profit.

The debate over whether the right to FoT should remain absolute will be driven by the public's desired trade-off between risk and freedom. However, there is a risk that the use of hard cases to frame this debate may lead reasoned argument to be overcome by political pressure. For example, emotions run understandably high in the situation of pedophiles and their potential risk to children. As a result, public sentiment that pedophiles should be punished for their thoughts alone could encourage legislators to make it permissible to violate the FoT of sex offenders (see *Doe v. City of Lafayette, Indiana*, 2004; Calvert, 2005; Human Rights Watch, 2007). As Calvert notes “It is easy to run for office and to support legislation when it is strategically and narrowly framed, such as the concise and visceral frame of “protect children from a pedophile” rather than the more complex and less emotionally appealing frame of “protect a constitutional right from legislative usurpation” (p. 130). If this Rubicon was crossed, with second-order sexual thoughts about children being punishable, other types of thought would inevitably be deemed suitable for thought punishment. This creates a “slippery slope” argument in favor of retaining the absolute status of the right to FoT. But should we abhor any attempt to limit the right to FoT because of what it may lead to, or is the risk of starting down such a slope one that needs to be taken (Volokh, 2003)?

There is also need to encourage the academic community to engage in the debate about the nature of the right to FoT. One barrier to this is the unchallenged promulgation of the idea that to take threats to FoT seriously is to engage in a “mental privacy panic” [Shen, 2013, p. 656]. Another is the perception that neural information, which could reveal someone's thoughts, could never be obtained without someone's knowledge and consent. For example, Smith (2013) notes that “you need a 15-ton, US$3-million fMRI machine and a person willing to lie very still inside it and actively think secret thoughts.” Similarly, Ryberg (2017) dryly notes that “one does not just end up in an MRI-scanner without knowing this” (p. 198).

There are two objections to this. First, wearable brain-reading technology will be developed. Once it is, it is highly likely to be enthusiastically embraced by society once commercially available, due to the convenience it will offer. People will queue up to have their brains read. Soon, as with cars and the internet, it could become effectively impossible to function in society without this technology. Second, even if one is skeptical of this threat, one cannot ignore the clear and present danger posed to FoT posed by behavior-reading. Our inner world is already in the process of being inferred, without anyone going near a scanner.

The academic community hence needs to think now about the legal and ethical implications of new technologies for the right to FoT. This process could mirror that in the artificial intelligence (AI) community, which is considering both contemporary ethical issues raised by AI, as well as the ethical issues it could raise in the future as AI technology further advances (e.g., Bostrom and Yudkowsky, 2014; Dignum, 2018).

### Wider Questions

We may also want to consider whether the culture we live in has shifted from that in which the ideal of the mentally autonomous, self-determining individual was born. The 1948 Universal Declaration of Human Rights granted people rights to achieve the culturally evolved ideal of being an autonomous individual (Zuboff, 2019). However, the rise of neoliberal market economics created a society which began to undermine the ability of people to be self-determining (Smail, 2018; Zuboff, 2019). Arguably, there is now a “yawning gap between the right of self-assertion and the capacity to control the social settings which render such self-assertion feasible” (Bauman, as cited in Zuboff, 2019, p. 45).

Not only may the ability for self-determination be ebbing, but so may the desire to think itself. Jacobs (2018) has argued that “Relatively few people *want* to think. Thinking troubles us; thinking tires us” (p. 17). There is some evidence for this. Consider a recent study by Wilson et al. (2014). This began by noting that the 2012 American Time Use Survey found that 83% of American adults spent no time at all “relaxing or thinking.” Could it be, Wilson and colleagues asked, that people do not enjoy thinking? To test this, they left college students in a room on their own for 15 minutes, without their belongings, and asked them “to spend the time entertaining themselves with their thoughts.” However, they were permitted one potential activity; they could press a button and get an electric shock. Two-thirds of men, and a quarter of women, gave themselves at least one electric shock in the 15 min period. How far have we come from the centrality of thought to human life, as stressed by the Founding Fathers of the United States and its most eminent Supreme Court Justices, when we would rather torture ourselves than think? Would we rather governments and corporations do our thinking for us, by serving up predictions and nudges for us to simply follow?

Other questions also arise along these lines. Have we morphed into a “culture of control” (Garland, 2001), more driven by the desire to prevent risk than preserve freedom? Has surveillance capitalism's interest in us being predictable rather than free impacted our view of ourselves (Zuboff, 2019)? How do we conceive what it is to be human today, and how does this conception map onto a right to FoT?

This paper hopes to stimulate a public debate on FoT, as well as interdisciplinary conversations between lawyers, neuroscientists, psychologists, philosophers and those working in the technology industries. Society needs to be structured to encourage and support citizens to be able to think in such debates. Adapting Balkin's (1990) arguments relating to free speech, there is a need to move away from simply considering judicial protection of “free thought rights” to a wider implementation of what we could call “free thought values” into the fabric of society and its institutions. In a time-poor society, in which everything, even opinions, need to be delivered on demand, we may ask where the space currently is for thought. This encourages rule-of-thumb thought. Rule-of-reason thought takes time and effort and hence risks becoming privileged. As Virginia Woolf put it “five hundred [pounds] a year stands for the power to contemplate” (p. 125). Governments need to give their citizens tools and time for thought, which they have a duty to do under the positive aspect of the right to FoT. As has been noted by the U.S. Supreme Court, without opportunities for “serenity and reflection… freedom of thought becomes a mocking phrase, and without freedom of thought, there can be no free society” (*Kovacs v. Cooper*, 1949). And yet, thinking is also a communal process. U.S Supreme Court Justice Brandeis was fond of quoting Isaiah 1:18, “Come now, and let us reason together” (Rosen, 2016). Thinking is something we must also do together, rather than just in isolation potentially based on for-your-eyes-only micro-targeted information.We hence need both public and private spaces for thought.

The fate of FoT is not just in governmental hands. It also depends on citizens. We must value free thought. We must be courageous, as mental autonomy comes at a price. To allow FoT, one must accept a degree of risk. The land of the free has to also be the home of the brave. The fate of FoT hence depend on both top-down governmental support and bottom-up popular support. Should we collectively stumble in our defense of humanity's right to FoT, the creature that gets to its feet again may not be recognizably human.

## Author Contributions

The author confirms being the sole contributor of this work and has approved it for publication.

### Conflict of Interest

The author declares that the research was conducted in the absence of any commercial or financial relationships that could be construed as a potential conflict of interest.

## References

[B1] Abood v. Detroit Board of Education (1977). 431 U.S. 209, 97 S. Ct. 1782, 52 L. Ed. 2d 261.

[B2] Ajzen I. (1991). The theory of planned behavior. Organizat. Behav. Hum. Decis. Process. 50, 179–211. 10.1016/0749-5978(91)90020-T

[B3] Alderson-Day B. Fernyhough C. (2015). Inner speech: development, cognitive functions, phenomenology, and neurobiology. Psychol. Bull. 141, 931–965. 10.1037/bul0000021 26011789 PMC4538954

[B4] Alegre S. (2017). Rethinking freedom of thought for the 21^st^ century. Eur. Hum. Rights Law Rev. 3, 221–233.

[B5] Anderson A. J. Binder J. R. Fernandino L. Humphries C. J. Conant L. L. Aguilar M.. (2016). Predicting neural activity patterns associated with sentences using a neurobiologically motivated model of semantic representation. Cereb. Cortex 27, 4379–4395. 10.1093/cercor/bhw240 27522069

[B6] Anderson C. Hildreth J. A. D. Howland L. (2015). Is the desire for status a fundamental human motive? A review of the empirical literature. Psychol. Bull. 141, 574–601. 10.1037/a0038781 25774679

[B7] Anumanchipalli G. K. Chartier J. Chang E. F. (2019). Speech synthesis from neural decoding of spoken sentences. Nature 568, 493–498. 10.1038/s41586-019-1119-1 31019317 PMC9714519

[B8] Ariely D. Berns G. S. (2010). Neuromarketing: the hope and hype of neuroimaging in business. Nat Rev Neurosci. 11, 284–292. 10.1038/nrn2795 20197790 PMC2875927

[B9] Asch S. E. (1952). Social Psychology. New York, NY: Prentice-Hall. 10.1037/10025-000

[B10] Ashcroft v. Free Speech Coalition (2002). 535 U.S. 234, 122 S. Ct. 1389, 152 L. Ed. 2d 403.

[B11] Austin J. (1885). Lectures on Jurisprudence. London: John Murray

[B12] Balkin J. M. (1990). Some realism about pluralism: legal realist approaches to the First Amendment. Duke Law J. 375–430. 10.2307/1372553

[B13] Baumeister R. F. Leary M. R. (1995). The need to belong: desire for interpersonal attachments as a fundamental human motivation. Psychol. Bull. 117, 497–529. 10.1037//0033-2909.117.3.497 7777651

[B14] Beck A. T. Haigh E. A. (2014). Advances in cognitive theory and therapy: the generic cognitive model. Annu. Rev. Clin. Psychol. 10, 1–24. 10.1146/annurev-clinpsy-032813-153734 24387236

[B15] Berns G. S. Chappelow J. Zink C. F. Pagnoni G. Martin-Skurski M. E. Richards J. (2005). Neurobiological correlates of social conformity and independence during mental rotation. Biol. Psychiatry 58, 245–253. 10.1016/j.biopsych.2005.04.012 15978553

[B16] Blackstone W. (1832). Commentaries on the Laws of England. Vol. 2. New York, NY: Collins and Hannay.

[B17] Blitz M. J. (2010). Freedom of thought for the extended mind: cognitive enhancement and the constitution. Wisconsin Law Rev. 2010:1049.

[B18] Boire R. G. (2001). On cognitive liberty. J. Cogn. Libert. 2, 7–22. 10.1016/S1297-9562(01)90048-8

[B19] Bond R. M. Fariss C. J. Jones J. J. Kramer A. D. Marlow C. Settle J. E.. (2012). A 61-million-person experiment in social influence and political mobilization. Nature 489, 295–298. 10.1038/nature11421 22972300 PMC3834737

[B20] Bostrom N. Yudkowsky E. (2014). The ethics of artificial intelligence, in The Cambridge Handbook of Artificial Intelligence, eds Frankish K. Ramsey W. M. (Cambridge: Cambridge University Press), 316–334. 10.1017/CBO9781139046855.020

[B21] Brandenburg v. Ohio (1969). 395 U.S. 444, 89 S. Ct. 1827, 23 L. Ed. 2d 430.

[B22] Branti v. Finkel (1980). 445 U.S. 507, 100 S. Ct. 1287, 63 L. Ed. 2d 574.

[B23] Brehm J. W. (1966). A Theory of Psychological Reactance. Oxford: Academic Press.

[B24] Brudner A. (2009). Punishment and Freedom. Oxford: Oxford University Press. 10.1093/acprof:oso/9780199207251.001.0001

[B25] Bublitz J. C. (2011). If man's true palace is his mind, what is its adequate protection? On a right to mental self-determination and limits of interventions into other minds, in Technologies on the Stand: Legal and Ethical Questions in Neuroscience and Robotics, eds van den Berg B. Klaming L. (Nijmegen: Wolf Legal Publishers, 95–121.

[B26] Bublitz J. C. (2013). My mind is mine!? Cognitive liberty as a legal concept, in Cognitive Enhancement, eds Hildt E. Fraknke A. G. (Dordrecht: Springer), 233–264. 10.1007/978-94-007-6253-4_19

[B27] Bublitz J. C. (2014). Freedom of thought in the age of neuroscience. Archiv Rechts-und Sozialphilosphie 100, 1–25.

[B28] Bublitz J. C. Merkel R. (2014). Crimes against minds: on mental manipulations, harms and a human right to mental self-determination. Crim. Law Philosophy 8, 51–77. 10.1007/s11572-012-9172-y

[B29] Byers E. S. Purdon C. Clark D. A. (1998). Sexual intrusive thoughts of college students. J. Sex Res. 35, 359–369. 10.1080/00224499809551954

[B30] Cadwalladr, C. (2018, March 19) ‘I made Steve Bannon's psychological warfare tool': meet the data war Whistleblower. *The Guardian*. Retrieved from https://www.theguardian.com/news/2018/mar/17/data-war-whistleblower-christopher-wylie-faceook-nix-bannon-trump.

[B31] Calvert C. (2005). Freedom of thought, offensive fantasies and the fundamental human right to hold deviant ideas: why the Seventh Circuit got it wrong in Doe v. City of Lafayette, Indiana. Pierce Law Rev. 3, 125–160.

[B32] Centre for Humane Technology (2019a). Design Guide. Available online at: https://humanetech.com/designguide/ (accessed August 01, 2019).

[B33] Centre for Humane Technology (2019b). Take Control. Available online at: https://humanetech.com/resources/take-control/ (accessed August 01, 2019).

[B34] Chatzisarantis N. L. Hagger M. S. Wang C. J. Thøgersen-Ntoumani C. (2009). The effects of social identity and perceived autonomy support on health behaviour within the theory of planned behaviour. Curr. Psychol. 28, 55–68. 10.1007/s12144-009-9043-4

[B35] Chomsky N. (1989). Necessary Illusions: Thought Control in Democratic Societies. London: Pluto Press.

[B36] Cialdini R. B. (2013). Influence: Science and Practice. New York, NY: Harper Collins.

[B37] Cialdini R. B. Goldstein N. J. (2004). Social influence: Compliance and conformity. Annu. Rev. Psychol. 55, 591–621. 10.1146/annurev.psych.55.090902.142015 14744228

[B38] Clark A. Chalmers D. (1998). The extended mind. Analysis 58, 7–19. 10.1111/1467-8284.00096

[B39] Cohen J. E. (2000). Examined lives: informational privacy and the subject as object. Stanford Law Rev. 52, 1373–1438. 10.2307/1229517

[B40] COMEST (2005). The Precautionary Principle. Paris: UNESCO. Retrieved 2 June 2019 from https://unesdoc.unesco.org/ark:/48223/pf0000139578

[B41] Corry R. J. Jr. (2000). Burn this article: it is evidence in your thought crime prosecution. Texas Rev. Law Polit. 4, 462–488.

[B42] Deibert R. J. (2019). The road to digital unfreedom: three painful truths about social media. J. Democr. 30, 25–39. 10.1353/jod.2019.0002

[B43] Denver Area Educational Telecommunications Consortium Inc. v. Federal Communications Commission. (1996). 518 U.S. 727, 802-03, 116 S. Ct. 2374, 135 L. Ed. 2d 888.

[B44] Dignum J. (2018). Ethics in artificial intelligence: introduction to the special issue. Ethics Inform. Technol. 20, 1–3. 10.1007/s10676-018-9450-z

[B45] Doe v. City of Lafayette Indiana. (2001). 160 F. Supp. 2d 996 (N.D. Ind.).

[B46] Doe v. City of Lafayette Indiana. (2004). 377 F.3d 757 (7th Cir.).

[B47] Doe v. City of Lafayette Indiana. (2003). 334 F.3d 606 (7th Cir.).

[B48] Duff R. A. (1998). Philosophy and the Criminal Law: Principle and Critique. Cambridge: Cambridge University Press. 10.1017/CBO9780511527371

[B49] Duff R. A. (2007). Answering for Crime: Responsibility and Liability in the Criminal Law. London: Bloomsbury Publishing. 10.1093/acprof:oso/9780199237159.003.0012

[B50] Eriksen C. W. Kuethe J. L. (1956). Avoidance conditioning of verbal behavior without awareness: A paradigm of repression. J. Abnorm. Soc. Psychol. 53, 203–209. 10.1037/h0040008 13357216

[B51] Eyal N. (2014). Hooked: How to Build Habit-Forming Products. London: Penguin UK.

[B52] Feinberg J. (1987). Harm to Others, Vol. 1. Oxford: Oxford University. 10.1093/0195046641.003.0001

[B53] Ferster C. B. Skinner B. F. (1957). Schedules of Reinforcement. East Norwalk, CT: Appleton-Century-Crofts. 10.1037/10627-000

[B54] *Folgerø and Others v. Norway* (2007). GC, 2 E.C.H.R.

[B55] Frankfurt H. G. (1971). Freedom of the will and the concept of a person. J. Philosophy 68, 5–20. 10.2307/2024717

[B56] Garland D. (2001). The Culture of Control: Crime and Social Order in Contemporary Society. Chicago, IL: University of Chicago Press. 10.7208/chicago/9780226190174.001.0001

[B57] Golbeck J. Robles C. Turner K. (2011). Predicting personality with social media, in CHI'11 Extended Abstracts on Human Factors in Computing Systems (New York, NY: ACM), 253–262. 10.1145/1979742.1979614

[B58] Gongola J. Scurich N. Quas J. A. (2017). Detecting deception in children: a meta-analysis. Law Hum. Behav. 41, 44–54. 10.1037/lhb0000211 27685642

[B59] Greenwald G. (2013, July 31). XKeyscore: NSA tool collects 'nearly everything a user does on the internet. The Guardian. Retrieved from https://www.theguardian.com/world/2013/jul/31/nsa-top-secret-program-online-data.

[B60] Greenwood D. J. (2017). Neofederalism: the surprising foundations of corporate constitutional rights. Univ. Illinois Law Rev. 2017, 163–222.

[B61] Halliburton C. M. (2009). How privacy killed Katz: a tale of cognitive freedom and the property of personhood as fourth amendment norm. Akron Law Rev. 42, 803–884.

[B62] Hanson T. L. Diaz-Botia C. A. Kharazia V. Maharbiz M. M. Sabes P. N. (2019). The “sewing machine” for minimally invasive neural recording. bioRxiv 578542. 10.1101/578542

[B63] Haxby J. V. Gobbini M. I. Furey M. L. Ishai A. Schouten J. L. Pietrini P. (2001). Distributed and overlapping representations of faces and objects in ventral temporal cortex. Science 293, 2425–2430. 10.1126/science.1063736 11577229

[B64] Haynes J. D. Sakai K. Rees G. Gilbert S. Frith C. Passingham R. E. (2007). Reading hidden intentions in the human brain. Curr. Biol. 17, 323–328. 10.1016/j.cub.2006.11.072 17291759

[B65] Hersh E. D. Schaffner B. F. (2013). Targeted campaign appeals and the value of ambiguity. J. Polit. 75, 520–534. 10.1017/S0022381613000182

[B66] Holtug N. (2002). The harm principle. Ethical Theory Moral Pract. 5, 357–389. 10.1023/A:1021328520077

[B67] Horikawa T. Tamaki M. Miyawaki Y. Kamitani Y. (2013). Neural decoding of visual imagery during sleep. Science 340, 639–642. 10.1126/science.1234330 23558170

[B68] Human Rights Watch (2007). No Easy Answers: Sex Offender Laws in the US. Retrieved from https://www.hrw.org/report/2007/09/11/no-easy-answers/sex-offender-laws-us

[B69] i360 (2019, June 13). The database. Retrieved from https://www.i-360.com/the-database/.

[B70] Ienca M. Andorno R. (2017). Towards new human rights in the age of neuroscience and neurotechnology. Life Sci. Soc. Policy 13:5. 10.1186/s40504-017-0050-1 28444626 PMC5447561

[B71] Jacobs A. (2018). How to Think: A Guide for the Perplexed. London: Profile Books.

[B72] Johnson R. Cureton A. (2019). Kant's Moral Philosophy, in The Stanford Encyclopedia of Philosophy, Spring 2019 Edn., ed Zalta E. N.. Retrieved from https://plato.stanford.edu/archives/spr2019/entries/kant-moral/

[B73] Jorgensen C. Lee D. D. Agabont S. (2003). Sub auditory speech recognition based on EMG signals, in Proceedings of the International Joint Conference on Neural Networks, 2003. Vol. 4 (New York, NY:IEEE), 3128–3133.

[B74] Kahneman D. (2011). Thinking, Fast and Slow. London: Penguin.

[B75] Kalla J. L. Broockman D. E. (2018). The minimal persuasive effects of campaign contact in general elections: evidence from 49 field experiments. Am. Polit. Sci. Rev. 112, 148–166. 10.1017/S0003055417000363

[B76] Kanai R. Feilden T. Firth C. Rees G. (2011). Political orientations are correlated with brain structure in young adults. Curr. Biol. 21, 677–680. 10.1016/j.cub.2011.03.017 21474316 PMC3092984

[B77] Kay K. N. Naselaris T. Prenger R. J. Gallant J. L. (2008). Identifying natural images from human brain activity. Nature 452, 352–355. 10.1038/nature06713 18322462 PMC3556484

[B78] Keskin C. Kim D. Chau B. Kim J. Koishida K. Shahid K. (2018). Changing an Application State Using Neurological Data. U.S. Patent No. 9,864,431. Retrieved from: https://patents.google.com/patent/US9864431B2/en

[B79] Kildare C. A. Middlemiss W. (2017). Impact of parents mobile device use on parent-child interaction: a literature review. Comput. Hum. Behav. 75, 579–593. 10.1016/j.chb.2017.06.003

[B80] Klucharev V. Hytönen K. Rijpkema M. Smidts A. Fernández G. (2009). Reinforcement learning signal predicts social conformity. Neuron 61, 140–151. 10.1016/j.neuron.2008.11.027 19146819

[B81] Knox J. H. (2011). The Ruggie Rules: applying human rights law to corporations, in The UN Guiding Principles on Business and Human Rights: Foundations and Implementation, ed Mares R. (Lieden: Martinus Nijhoff Publishers), 51–83. 10.1163/9789004225794_003

[B82] Kokkinakis v. Greece (1994). 17 E.H.R.R. 397.

[B83] Kolber A. J. (2016). Two views of First Amendment thought privacy. J. Const. Law 18, 1381–1423.

[B84] *Kovacs v. Cooper* (1949). 336 U.S. 77, 69 S. Ct. 448, 93 L. Ed. 513.

[B85] Kosinski M. Stillwell D. Graepel T. (2013). Private traits and attributes are predictable from digital records of human behavior. Proc. Natl. Acad. Sci. U.S.A. 110, 5802–5805. 10.1073/pnas.1218772110 23479631 PMC3625324

[B86] Kramer A. D. Guillory J. E. Hancock J. T. (2014). Experimental evidence of massive-scale emotional contagion through social networks. Proc. Natl. Acad. Sci. U.S.A. 111, 8788–8790. 10.1073/pnas.1320040111 24889601 PMC4066473

[B87] Kruglanski A. W. Jasko K. Chernikova M. Milyavsky M. Babush M. Baldner C.. (2015). The rocky road from attitudes to behaviors: charting the goal systemic course of actions. Psychol. Rev. 122, 598–620. 10.1037/a0039541 26192134

[B88] Kuss D. J. Griffiths M. D. (2017). Social networking sites and addiction: ten lessons learned. Int. J. Environ. Res. Public Health 14:311. 10.3390/ijerph14030311 28304359 PMC5369147

[B89] LaPiere R. T. (1934). Attitudes vs. actions. Soc. Forces 13, 230–237. 10.2307/2570339

[B90] Larissis and Others v. Greece (1998). 65 Eur. Ct. H.R. (ser. A) 363, 27 E.H.R.R. 329.

[B91] Lewis, P. (2018, February 2). 'Fiction is outperforming reality': how YouTube's algorithm distorts truth. *The Guardian*. Retrieved from https://www.theguardian.com/technology/2018/feb/02/how-youtubes-algorithm-distorts-truth.

[B92] Liberini F. Redoano M. Russo A. Cuevas A. Cuevas R. (2018). Politics in the Facebook Era. Evidence from the 2016 US Presidential Elections. CAGE Online Working Paper Series (389).

[B93] Long Beach City Employees Assn. v. City of Long Beach (1986). 719 P.2d 660, 41 Cal. 3d 937, 227 Cal. Rptr. 90.

[B94] Loucaides L. G. (2012). The right to freedom of thought as protected by the European Convention on Human Rights. Cyprus Hum. Rights Law Rev. 1, 79–87.

[B95] Madison J. (1785). Memorial and Remonstrance Against Religious Assessments. Retrieved on 01-08-19 from https://founders.archives.gov/documents/Madison/01-08-02-0163

[B96] Marsh, S. (2018, January 1). Neurotechnology, Elon Musk and the goal of human enhancement. *The Guardian*. Retrieved from https://www.theguardian.com/technology/2018/jan/01/elon-musk-neurotechnology-human-enhancement-brain-computer-interfaces.

[B97] Marteau T. M. Ogilvie D. Roland M. Suhrcke M. Kelly M. P. (2011). Judging nudging: can nudging improve population health? Br. Med. J. 342:d228. 10.1136/bmj.d228 21266441

[B98] Marthews A. Tucker C. E. (2017). Government Surveillance and Internet Search Behavior. Available online at: https://ssrn.com/abstract=2412564

[B99] Martin S. Brunner P. Holdgraf C. Heinze H. J. Crone N. E. Rieger J.. (2014). Decoding spectrotemporal features of overt and covert speech from the human cortex. Front. Neuroeng. 7:14. 10.3389/fneng.2014.00014 24904404 PMC4034498

[B100] McCarthy-Jones S. (2019, February 28) Unwanted unacceptable thoughts: most people have them and we should talk about them. The Conversation. Retrieved from https://theconversation.com/unwanted-unacceptable-thoughts-most-people-have-them-and-we-should-talk-about-them-112245.

[B101] Mendlow G. S. (2018). Why is it wrong to punish thought. Yale Law J. 127, 2342–2387.

[B102] Metzinger T. (2015). M-autonomy. J. Conscious. Stud. 22, 270–302.

[B103] Metzinger T. K. (2013). The myth of cognitive agency: subpersonal thinking as a cyclically recurring loss of mental autonomy. Front. Psychol. 4:931. 10.3389/fpsyg.2013.00931 24427144 PMC3868016

[B104] Mill J. S. (1859/1985). On Liberty. London: Penguin.

[B105] Mirkovic B. Debener S. Jaeger M. De Vos M. (2015) Decoding the attended speech stream with multi-channel EEG: implications for online, daily-life applications. J. Neural Eng. 12, 046007. 10.1088/1741-2560/12/4/046007 26035345

[B106] Miyawaki Y. Uchida H. Yamashita O. Sato M. A. Morito Y. Tanabe H. C.. (2008). Visual image reconstruction from human brain activity using a combination of multiscale local image decoders. Neuron 60, 915–929. 10.1016/j.neuron.2008.11.004 19081384

[B107] Morris H. (1965). Punishment for thoughts. Monist 49, 342–376.

[B108] Mullender R. (2000). Theorizing the Third way: Qualified consequentialism, the proportionality principle, and the new social democracy. J. Law Soc. 27, 493–516. 10.1111/1467-6478.00165

[B109] Nagel T. (1998). Concealment and exposure. Philos. Public Affairs 27, 3–30. 10.1111/j.1088-4963.1998.tb00057.x

[B110] Naselaris T. Prenger R. J. Kay K. N. Oliver M. Gallant J. L. (2009). Bayesian reconstruction of natural images from human brain activity. Neuron 63, 902–915. 10.1016/j.neuron.2009.09.006 19778517 PMC5553889

[B111] Nishimoto S. Vu A. T. Naselaris T. Benjamini Y. Yu B. Gallant J. L. (2011). Reconstructing visual experiences from brain activity evoked by natural movies. Curr. Biol. 21, 1641–1646. 10.1016/j.cub.2011.08.031 21945275 PMC3326357

[B112] Nowak M. (1993). UN Covenant on Civil and Political Rights: CCPR Commentary. Kehl am Rhein: N. P. Engel.

[B113] Nuffield Council on Bioethics (2002). Genetics and Human Behaviour: The Ethical Context. London: Nuffield Council on Bioethics, London. Retrieved on 31 May 2019 from http://nuffieldbioethics.org/wp-content/uploads/2014/07/Genetics-and-human-behaviour.pdf

[B114] Olmstead v. United States (1928). 277 U.S. 438, 48 S. Ct. 564, 72 L. Ed. 944.

[B115] Pandey E. (2017, Nov 9). Sean Parker: Facebook was Designed to Exploit Human “vulnerability”. Retrieved from https://www.axios.com/sean-parker-facebook-was-designed-to-exploit-human-vulnerability-1513306782-6d18fa32-5438-4e60-af71-13d126b58e41.html.

[B116] Paxton J. M. Ungar L. Greene J. D. (2012). Reflection and reasoning in moral judgment. Cogn. Sci. 36, 163–177. 10.1111/j.1551-6709.2011.01210.x 22049931

[B117] Pereira F. Lou B. Pritchett B. Ritter S. Gershman S. J. Kanwisher N.. (2018). Toward a universal decoder of linguistic meaning from brain activation. Nat. Commun. 9, 963. 10.1038/s41467-018-03068-4 29511192 PMC5840373

[B118] Purdon C. Clark D. A. (1993). Obsessive intrusive thoughts in nonclinical subjects. Part I. Content and relation with depressive, anxious and obsessional symptoms. Behav. Res. Ther. 31, 713–720. 10.1016/0005-7967(93)90001-B 8257402

[B119] Rennie v. Klein (1981). 653 F.2d 836 (3d Cir.). 11648464

[B120] Rentfrow P. J. Gosling S. D. (2003). The do re mi's of everyday life: the structure and personality correlates of music preferences. J. Pers. Soc. Psychol. 84, 1236–1256. 10.1037/0022-3514.84.6.1236 12793587

[B121] Richards N. (2015). Intellectual Privacy: Rethinking Civil Liberties in the Digital Age. Oxford: Oxford University Press.

[B122] Richmond S. (2012). Brain imaging and the transparency scenario, in I Know What You're Thinking: Brain Imaging and Mental Privacy, eds Richmond S. Rees G. Edwards S. J. (Oxford: Oxford University Press), 185–204. 10.1093/acprof:oso/9780199596492.001.0001

[B123] Rosen J. (2016). Louis D. Brandeis: American Prophet. New Haven, CT: Yale University Press.

[B124] Ryberg J. (2017). Neuroscience, mind reading and mental privacy. Res. Publica 23, 197–211. 10.1007/s11158-016-9343-0

[B125] Ryssdal, K., Garrova, R. (2017, April 25) ‘The Circle' author Dave Eggers thinks the internet is getting creepier. *Marketplace*. Retrieved from: https://www.marketplace.org/2017/04/25/tech/circle-author-dave-eggers-thinks-internet-getting-creppier/

[B126] Schreiber D. Fonzo G. Simmons A. N. Dawes C. T. Flagan T. Fowler J. H.. (2013). Red brain, blue brain: evaluative processes differ in Democrats and Republicans. PLoS ONE 8:e52970. 10.1371/journal.pone.0052970 23418419 PMC3572122

[B127] Schüll N. D. (2014). Addiction by Design. Princeton, NJ: Princeton University Press.

[B128] Shen F. X. (2013). Neuroscience, mental privacy, and the law. Harv. J. Law Public Policy 36, 653–714.

[B129] Shultz S. Dunbar R. I. M. (2012). The social brain hypothesis, in I Know What You're Thinking: Brain Imaging and Mental Privacy, eds Richmond S. Rees G. Edwards S. J. (Oxford: Oxford University Press, 13–28.

[B130] Smail D. (2018). The Origins of Unhappiness: A New Understanding of Personal Distress. London: Routledge. 10.4324/9780429482632

[B131] Smith K. (2013). Brain decoding: reading minds. Nat. News. 502, 428–430. 10.1038/502428a 24153277

[B132] Solon O. (2017, April 19). Facebook has 60 people working on how to read your mind. The Guardian, Retrieved from https://www.theguardian.com/technology/2017/apr/19/facebook-mind-reading-technology-f8.

[B133] Soon C. S. Brass M. Heinze H. J. Haynes J. D. (2008). Unconscious determinants of free decisions in the human brain. Nat. Neurosci. 11, 543–545. 10.1038/nn.2112 18408715

[B134] Stanley v. Georgia (1969). 394 U.S. 557, 89 S. Ct. 1243, 22 L. Ed. 2d 542.

[B135] State of Washington v. Stevenson (2005). 189 128 Wn. App. 179.

[B136] Steffan v. Perry (1994). 41 F.3d 677, 713-14 (D.C.Cir.).

[B137] Sumption J. (2019). The Reith Lectures 2019/ Law and the Decline of Politics. Lecture 1: Law's Expanding Empire. Available online at: http://downloads.bbc.co.uk/radio4/reith2019/Reith_2019_Sumption_lecture_1.pdf

[B138] Thaler R. Sunstein C. (2008). Nudge. Improving Decisions About Health, Wealth and Happiness. New Haven, CN: Yale University Press.

[B139] U.S. v. Bredimus (2003). 352 F.3d 200, 207-08 (5th Cir.).

[B140] U.S. v. Gamache (1998). 156 F.3d 1, 8 (1st Cir.).

[B141] U.S. v. Kaechele (2006). 466 F. Supp. 2d 868 (E.D. Mich.).

[B142] U.S. v. Price (1998). 134 F.3d 340, 351 (6th Cir.).

[B143] U.S. v. Stokes (2013). 726 F.3d 880 (7th Cir.).

[B144] U.S. v. Tykarsky (2006). 446 F.3d 458 (3d Cir.).

[B145] United Nations (2011). Guiding Principles on Business and Human Rights: Implementing the United Nations“Protect, Respect and Remedy” Framework. Geneva: United Nations. Retrieved from https://www.ohchr.org/Documents/Publications/GuidingPrinciplesBusinessHR_EN.pdf

[B146] United Nations Human Rights Committee (1993). General Comment No. 22: The Right to Freedom of Thought, Conscience and Religion (Art. 18) (Geneva). Available online at: https://www.refworld.org/docid/453883fb22.html

[B147] United Nations Human Rights Committee (2004). General Comment No. 31: The Nature of the General Legal Obligation Imposed on States Parties to the Covenant (Geneva). Available online at: https://www.refworld.org/docid/478b26ae2.html

[B148] United Nations Human Rights Committee (2011). General Comment 34 on Article 19 of the ICCPR on Freedoms of Opinion and Expression, UN Doc CCPR/C/GC/34. Available online at: https://www.refworld.org/docid/453883fb22.html

[B149] van den Berg B. (2011). Robots as tools for techno-regulation. Law Innovat. Technol. 3, 319–334. 10.5235/175799611798204905

[B150] van den Berg B. Leenes R. (2011). Keeping up appearances: audience segregation in social network sites, in Computers, Privacy and Data Protection: An Element of Choice, eds Gutwirth S. Poullet Y. de Hert P. Leenes R. (Dordrecht: Springer), 211–231. 10.1007/978-94-007-0641-5_10

[B151] van den Berg B. Leenes R. E. (2013). Abort, retry, fail: scoping techno-regulation and other techno-effects, in Human Law and Computer Law: Comparative Perspectives, eds Hildebrandt M. Gaakeer J. (Heidelberg: Springer), 67–87. 10.1007/978-94-007-6314-2_4

[B152] Veale D. Freeston M. Krebs G. Heyman I. Salkovskis P. (2009). Risk assessment and management in obsessive–compulsive disorder. Adv. Psychiatr. Treat. 15, 332–343. 10.1192/apt.bp.107.004705

[B153] Vermeulen B. (2006). Freedom of thought, conscience and religion (article 9), in Theory and Practice of the European Convention on Human Rights, 4th Edn., eds van P. Dijk F. van Hoof A. van Rijn Zwaak L. (Cambridge: Intersentia Press), 751–772.

[B154] Volokh E. (2003). The mechanisms of the slippery slope. Harvard Law Rev. 116, 1026–1137. 10.2307/1342743

[B155] Vygotsky L. S. (1978). Mind in Society: The Development of Higher Mental Processes, eds Cole M. John-Steiner V. Scribner S. Souberman E. (Cambridge, MA: Harvard University Press).

[B156] Walster E. Festinger L. (1962). The effectiveness of “overheard” persuasive communications. J. Abnorm. Soc. Psychol. 65, 395–402. 10.1037/h0041172 13998661

[B157] Wang J. Cherkassky V. L. Just M. A. (2017). Predicting the brain activation pattern associated with the propositional content of a sentence: modeling neural representations of events and states. Hum. Brain Mapp. 38, 4865–4881. 10.1002/hbm.23692 28653794 PMC6867144

[B158] Wang Y. Kosinski M. (2018). Deep neural networks are more accurate than humans at detecting sexual orientation from facial images. J. Pers. Soc. Psychol. 114, 246–257. 10.1037/pspa0000098 29389215

[B159] Warren S. D. Brandeis L. D. (1890). Right to privacy. Harvard Law Rev. 4:193. 10.2307/1321160

[B160] Washington v. Harper (1990). 494 U.S. 210, 110 S. Ct. 1028, 108 L. Ed. 2d 178.

[B161] Wellman H. M. Cross D. Watson J. (2001). Meta-analysis of theory-of-mind development: the truth about false belief. Child Dev. 72, 655–684. 10.1111/1467-8624.00304 11405571

[B162] Wells A. Davies M. I. (1994). The Thought Control Questionnaire: A measure of individual differences in the control of unwanted thoughts. Behav. Res. Ther. 32, 871–878. 10.1016/0005-7967(94)90168-6 7993332

[B163] Westin A. F. (1967). Privacy and Freedom. New York, NY: Atheneum.

[B164] Whalen v. Roe (1977). 429 U.S. 589, 97 S. Ct. 869, 51 L. Ed. 2d 64.

[B165] Wicker A. W. (1969). Attitudes versus actions: The relationship of verbal and overt behavioral responses to attitude objects. J. Soc. Issues 25, 41–78. 10.1111/j.1540-4560.1969.tb00619.x

[B166] Wilson T. D. Reinhard D. A. Westgate E. C. Gilbert D. T. Ellerbeck N. Hahn C.. (2014). Just think: the challenges of the disengaged mind. Science 345, 75–77. 10.1126/science.1250830 24994650 PMC4330241

[B167] Woolf V. (2001). A Room of One's Own. Ontario, ON: Broadview Press.

[B168] Yu R. Sun S. (2013). To conform or not to conform: spontaneous conformity diminishes the sensitivity to monetary outcomes. PLoS ONE 8:e64530. 10.1371/journal.pone.0064530 23691242 PMC3656845

[B169] Yuste R. Goering S. Bi G. Carmena J. M. Carter A. Fins J. J.. (2017). Four ethical priorities for neurotechnologies and AI. Nat. News 551, 159–163. 10.1038/551159a 29120438 PMC8021272

[B170] Zuboff S. (2019). The Age of Surveillance Capitalism. London: Profile Books.

