# Corrigendum: The Autonomous Mind: The Right to Freedom of Thought in the Twenty-First Century

**DOI:** 10.3389/frai.2021.672279

**Published:** 2021-04-15

**Authors:** Simon McCarthy-Jones

**Affiliations:** Department of Psychiatry, Trinity College Dublin, Dublin, Ireland

**Keywords:** human rights, privacy, psychology, law, machine learning, big data

An addition has been made to the **Acknowledgments**. The updated **Acknowledgments** statement appears below:

## Acknowledgments

“The author is grateful to the two reviewers of this paper, as well as Richard Mullender, Brendan Kelly, Patrick O'Callaghan, and Roseline McCarthy-Jones, for their insightful comments on this manuscript. The author would like to acknowledge Ms. Susie Alegre for introducing him to the right to freedom of thought in international law as applied to contemporary technological developments, including the work of Vermeulen (2006), Bublitz (2014), Bublitz and Merkel (2014) and Boire (2001). This framework was applied in a separate analysis in Alegre (2017).”

The author states that this does not change the scientific conclusions of the article in any way. The original article has been updated.
